# Risk Factors for Urological Complications Associated with Caesarean Section—A Case-Control Study

**DOI:** 10.3390/medicina58010123

**Published:** 2022-01-14

**Authors:** Viorel Dragos Radu, Anda Ioana Pristavu, Angela Vinturache, Pavel Onofrei, Demetra Gabriela Socolov, Alexandru Carauleanu, Lucian Boiculese, Sadyie Ioana Scripcariu, Radu Cristian Costache

**Affiliations:** 1 Department of Urology, Faculty of Medicine, University of Medicine and Pharmacy “Gr. T. Popa”, 700115 Iasi, Romania; vioreldradu@yahoo.com (V.D.R.); onofrei.pavel@gmail.com (P.O.); criscostache@hotmail.com (R.C.C.); 2 Urological Department, “C.I. Parhon” University Hospital, 700115 Iasi, Romania; 3 Department of Obstetrics and Gynaecology, Faculty of Medicine, University of Medicine and Pharmacy “Gr. T. Popa”, 700115 Iasi, Romania; socolov@hotmail.com (D.G.S.); acarauleanu@yahoo.com (A.C.); isscripcariu@gmail.com (S.I.S.); 4 Obstetrics and Gynaecology Department, “Cuza Voda” Obstetrics and Gynecology University Hospital, 700115 Iasi, Romania; 5 Department Obstetrics & Gynecology, Queen Elizabeth II Hospital, Grande Prairie, AB T8V 2E8, Canada; angela.vinturache@gmail.com; 6 Department of Medical Informatics and Biostatistics, Faculty of Medicine, University of Medicine and Pharmacy “Gr. T. Popa”, 700115 Iasi, Romania; lboiculese@gmail.com

**Keywords:** urologic complications, bladder injury, ureter injury, caesarean hysterectomy, placenta accreta, placenta previa

## Abstract

*Background and Objectives:* Acute urologic complications, including bladder and/or ureteric injury, are rare but known events occurring at the time of caesarean section (CS). Delayed or inadequate management is associated with increased morbidity and poor long-term outcomes. We conducted this study to identify the risk factors for urologic injuries at CS in order to inform obstetricians and patients of the risks and allow management planning to mitigate these risks. *Materials and Methods:* We reviewed all cases of urological injuries that occurred at CS surgeries in a tertiary university centre over a period of four years, from January 2016 to December 2019. To assess the risk factors of urologic injuries, a case-control study of women undergoing caesarean delivery was designed, matched 1:3 to randomly selected women who had an uncomplicated CS. Electronic medical records and operative reports were reviewed for socio-demographic and clinical information. Descriptive and univariate analyses were used to characterize the study population and identify the risk factors for urologic complications. *Results:* There were 36 patients with urologic complications out of 14,340 CS patients, with an incidence of 0.25%. The patients in the case group were older, had a lower gestational age at time of delivery and their newborns had a lower birth weight. Prior CS was more prevalent among the study group (88.2 vs. 66.7%), as was the incidence of placenta accreta and central praevia. In comparison with the control group, the intraoperative blood loss was higher in the case group, although there was no difference among the two groups regarding the type of surgery (emergency vs. elective), uterine rupture, or other obstetrical indications for CS. Prior CS and caesarean hysterectomy were risk factors for urologic injuries at CS. *Conclusions:* The major risk factor for urological injuries at the time of CS surgery is prior CS. Among patients with previous CS, those who undergo caesarean hysterectomy for placenta previa central and placenta accreta are at higher risk of surgical haemostasis and complex urologic injuries involving the bladder and the ureters.

## 1. Introduction

Lower urinary tract injuries at the time of caesarean section (CS) can be divided in two categories: bladder injuries, with reported rates between 0.13% and 0.44% [1,2,3,4,5,6,7,8], and ureteral injuries, which are rarer, with reported rates between 0.01% and 0.08% [1,2,4,5,8,9]. Although intuitive for most practitioners, the risk of lower urinary tract injury at the time of CS has not been thoroughly investigated, with most of the evidence coming from small case series. A few retrospective cohort and case-control studies have returned conflicting findings, owing mostly to the inconsistent definitions of the injuries and lack of details of the extension and severity of the damage.

Urological injuries pose challenges in being recognized at the time of surgery and have the potential to create great postoperative distress to both patients and health care providers [9,10]. While most bladder injuries are easier recognized and solved intraoperatively, ureteric injuries are diagnosed late and, if recognized, generally require the presence of a specialist urologist in the operative field [2,11,12,13], which is not always feasible. Early recognition and repair of lower urinary tract injuries during CS is essential for optimal patient outcome and the prevention of late complications such as kidney damage and genitourinary fistula. Furthermore, the management of ureteric injuries diagnosed postoperatively is still controversial. 

Most previous studies assessed bladder injuries at the time of CS and identified several risk factors for this type of damage, including prelabour emergent delivery, caesarean delivery in second stage, attempted vaginal birth after CS, uterine rupture, adhesions, and increased body mass index [3]. The evidence of ureteral injury at the CS is scarce, with very few studies addressing this topic, despite the severity of such complications. Therefore, we conducted a retrospective case-control study, in which we aimed to identify the risk factors that forecast urological complications, bladder and ureteric, at the time of CS. We also present, with informative titles, the type and the characteristics of the urological injuries that appeared during CS, the time of recognition, and the repair techniques used. 

## 2. Materials and Methods

The study population comprised all pregnant women who underwent delivery by CS between January 2016 and December 2019 at a large tertiary maternity centre affiliated with a major medical university in Romania. A total of 25,278 deliveries were recorded in the maternity centre during the above time interval, of which 14,340 (56.72%) were by CS. Among these, 36 women had a diagnosis of urological injury at the time of surgical delivery, and they were included in the case group. These were matched 1:3 to a group of women randomly selected from the remaining pool of women who delivered by CS. A total of 102 women who had an uncomplicated CS were included in the control group.

In the study group were included all consecutive patients with a urological injury diagnosed at the time of CS delivery, or before the discharge from the hospital, several days to one week later. The urological complications analysed in this study included both bladder and ureteral injuries and were defined as laceration, total or partial transection, rupture of the bladder, and laceration, total or partial ligation, total of partial transection of the ureter, diagnosed either during surgery or after the surgery as hydronephrosis or leakage of contrast at radiological investigations. Patients with delayed recognition of urologic injuries or those who developed urogenital fistula or hydronephrosis by ureteral ligation after hospital discharge were excluded from the study. The control group included randomly selected women who had a surgical delivery with an uncomplicated CS. 

The cases were identified from the medical records using the ICD 10 codes for injury of the ureter (S37.1), bladder (S37.2), and urethra (S37.3). Information on demographics, socioeconomic status, and obstetrical and surgical events during pregnancy and at delivery were abstracted from the electronic medical records. Ambulatory outpatient records retrieved from the Urology Clinic associated with the university hospital, where women would have received further management for urologic injuries, were also consulted. This study received ethics approval from the ethics committee of Cuza-Voda University Hospital and C.I. Parhon University Hospital.

The sociodemographic variables studied were maternal age, education, place of residence, and socioeconomic status. The obstetrical variables were gravidity, parity, gestational age at the time of delivery, type of labour (spontaneous vs. induced), characteristics of labour and delivery, type of CS (emergency, elective, caesarean hysterectomy), indication for CS, status of chorionic membranes at the time of CS (ruptured or intact), foetal position, presence of abnormal placentation (placenta accreta, placenta previa), and presence of uterine rupture. 

The newborn variables collected included Apgar score and birth weight. Other clinical variables included were seniority of the surgeon (specialist vs. consultant), number of previous CS, type of uterine incision, type of anaesthesia, pre-existing maternal health conditions, previous surgery with or without known perioperative adhesions, estimated intraoperative blood loss, number of blood transfusion units, presence of adhesions, and additional surgical procedures performed at the time of CS (total or subtotal hysterectomy, adnexectomy, hypogastric arteries ligation, adhesiolysis). The term of intraabdominal adhesions refers to post-procedural or post-infective adhesions of the abdominal wall, bladder, and intestine to the uterus, omentum, and adhesive bands, without intestinal obstruction. For urologic injury cases, additional information about injury was collected, including injury location, time when it was recognized, time of injury (if documented), size of injury, presence of ureteral injury (associated or isolated), need for stent placement, type of repair performed, and surgical outcomes. Data collected were imputed into an Excel sheet and then imported into the statistical software for further data cleaning and analysis. The main outcome of this study was to identify the risk factors of urological injuries at the time of surgical delivery. The secondary outcome was to assess the types and characteristics of urological complications that occurred at CS.

## 3. Statistical Analysis

### 3.1. Sample Calculation

A sample size calculation was performed considering a type 1 error α = 0.05, an acceptable power of 0.80 (1-β), a proportion of approximately 0.4 of previous cessation from women undergoing CS, and an odds ratio of 3.0, assumed based on previous published evidence of a threefold risk increase of bladder injury in the group of women with prior CS compared to women with no prior CS. For a study group of 36, which was the number of women diagnosed with urologic injuries, a control sample of 101–108 subjects was required for meaningful statistical comparisons. Therefore, we chose the proportion of cases to controls to be approximately 1 to 3. The final control group size was represented by 102 women who underwent uncomplicated CS. The G*Power (version 3.1.9.6, Heinrich Heine University, Düsseldorf, Germany) program was used for sample size calculation. 

### 3.2. Statistical Tests

Descriptive statistics were used to describe all study variables. Means, standard deviations, and percentages were used to analyse continuous data, and frequency distributions were used to analyse categorical data. For numerical data, normality distribution was checked with the Kolmogorov-Smirnov test. Univariate analyses examined the associations between variables. The Mann-Whitney U test, chi-square, and Fisher’s exact test were used for categorical variables, and the Student’s T test for continuous variables, as indicated. Multivariable logistic regression analysis was performed to assess the risk factors for urological injuries at CS. Odds ratios (ORs) were calculated with 95% confidence intervals (CIs), and all associations were considered statistically significant at *p* < 0.05. SPSS version 23 (IBM SPSS, Chicago, IL, USA) was used for statistical analysis. Due to low incidence of various types of urologic injuries, the multivariable regression did not provide meaningful comparisons, and the findings are not included in Section 4. 

## 4. Results

During the 4-year time interval between January 2016 and December 2019, 14,340 women delivered by CS at out tertiary maternity hospital. Among the women who underwent delivery by CS within the study time frame, 36 women were identified with injuries of the urinary tract, representing an overall incidence of 0.25% from all CS performed. When stratified by type of urological injury, 32 women (88.8%) had bladder injuries and four women (11.1%) had injuries of the ureter.

Table 1 shows the demographic and clinical characteristics of cases and controls. Women who had urologic injuries were older, 32.4 vs. 28.8 years, and had a greater parity, 3.4 on average vs. 2.2, and a higher BMI, 29.9 vs. 27.9 kg/m^2^, than controls. There were no further differences between the two groups regarding other demographic characteristics. The newborns in the control group had a more advanced gestational age at the time of delivery and a higher birthweight. There were no differences in the Apgar scores between the two groups. 

Data are presented as mean ± SD and median (IQR) for continuous and *n* (%) for categorical variables.

Pre-existing medical conditions include cardiac, endocrinologic, and gastroenterological conditions, diabetes mellitus, and hearing loss. 

The characteristics of CS in the two groups are described in Table 2. Women who had urologic injuries were more likely to have a repeat CS, intraabdominal adhesions, and a higher estimated blood loss at the time of surgery. There were also differences between the groups with regard to the indications for the CS. Women with urologic injury were more likely to have a CS for placenta accreta spectrum disorders and complete placenta previa, whereas marginal placenta previa, foetal distress, and foetopelvic disproportion were more common indications in women with uncomplicated CS. There was only one case of complete placenta praevia and no cases of placenta accreta in the control group. Caesarean hysterectomy and ligature of hypogastric arteries were more common in the group of women with urologic injuries.

Table 3 summarizes the characteristics of the urologic injuries, including the type and mechanism of injury, time of diagnosis, and management. Most injuries were suspected and diagnosed at the time of their occurrence (34/36, 94.4%), with direct involvement of a specialist urologist called for assistance in 32 of the cases. All bladder injuries (32) were diagnosed at the time of CS and repaired intraoperatively. The injuries involved bladder dome or the trigone, the majority of which occurred during the creation of the bladder flap (78.1%). Six injuries (18.7%) occurred during uterine incision or delivery of the foetus entry to the peritoneal cavity, and in one case (3.12%) the bladder was accidently included in the uterine suture. Among the bladder injuries, 21 cases were injuries of the posterior wall, over 5 cm in length. In 10 cases, the injury was prolonged to the anterior wall. Four cases had incomplete bladder wall injury, without affecting the bladder mucosa layer. Intraoperative haematuria raised the suspicion of bladder injury in eight patients. In one case, urine leakage through the fistula appeared 5 days after surgery. Cystoscopy was performed after surgery in two cases. 

Four patients had ureteral injury, of which two were recognized intraoperatively, but only in one case the urologist was present during surgery. In this last case, there was total ligature of the left ureter, which required follow-up surgery with ureteral reimplantation. Two other cases were diagnosed postoperatively. In one case, a pelvic haematoma formed, with subsequent compression of the pelvic left ureter. A double J stenting was inserted, which was kept in place for 3 months. The other case was a partial, bilateral ligature of pelvic ureters, which was solved with bilateral double J stenting for 3 months. 

From the 36 women with urological complications, a hysterectomy was performed in 27 cases, all of which had previous CS. The indications for hysterectomy were placenta accreta (21 cases), uterine atony (five cases), and uterine rupture (one case). In six women who underwent hysterectomy, the diagnosis of abnormal placentation was made intraoperatively, and 15 had suspicion of abnormally invasive placenta before surgery. 

## 5. Discussion

This study examined the incidence, risk factors, and characteristics of the urologic injuries occurring during CS deliveries in the setting of one tertiary academic centre. To date, there are only a handful of studies of urologic complications associated with CS, the majority of which are case series [6,7]. Despite a major increase in the number of CS in most centres worldwide, the incidence of this type of lesions remains relatively low and unchanged. We report a low overall incidence of urologic injuries, only 0.25%. This may be due to the rarity of these events, high obstetrical volume of our institution, as well as the surgical risk evaluation and assignment of repeat CS with increased risk to more experienced obstetricians (specialist or consultant). Other studies report an incidence similar with ours, between 0.28% [3] and 0.30% [14], although the former study reports only bladder injuries.

In agreement with previously published data, we did find an association between patient age at the time of CS and the risk of lower urinary tract injury. This finding is intuitive in the context of our study, as these injuries occurred predominantly for repeat CS and thus in older women. While BMI has been cited previously as a risk factor, this variable was not robust in our analyses, likely due to the small number of patients in our cohort with a BMI over 30. However, we did find higher rates of bladder injuries occurring among women who have undergone a prior CS. This is consistent with published data as well as with generalized knowledge regarding the altered anatomy of the vesicovaginal space following prior dissection in this area. As shown in other studies [3,15], we demonstrate that previous CS is the main risk factor for urologic injuries at the time of CS surgery. We report that previous CS increases the risk of all types of urological complications by four times. Contrary to other studies, we did not find this risk to be greater when CS was performed in an emergency as compared to an elective surgery [3,16,17]. The reason for this may reside in the fact that the vast majority of patients with potential risk factors underwent elective surgery [8]. In our study, most of the complex urological complications, particularly ureteral lesions, occurred in surgeries where CS was followed by hysterectomy due to morbidly adherent placenta, haemorrhage at the time of CS, or complete placenta previa, which carried a great risk of accreta [18,19,20]. In our study, 12 of the 21 patients with accreta were diagnosed in patients with complete placenta previa. Some of previous published studies excluded from their analyses the cases of caesarean hysterectomy, considering the risk for urological complications is escalated by the second procedure, hysterectomy [6,14]. It is our opinion that a study of CS complications should take into account all potential risk factors and the relationship between them [21,22]. Of note, we performed a hysterectomy in all patients diagnosed with placenta accreta. On the other hand, studies of the risk factors for lower urinary tract injury at the time of hysterectomy for benign reasons in non-pregnant patients found that bladder injury during surgery is associated with a prior history of CS [23]. 

Placenta accreta spectrum (PAS) is the most common cause of urological lesions after CS in the literature. PAS is frequently associated with placenta previa and invasion of the placenta into the bladder. The anatomy is frequently distorted, and there is frequently abundant neovascularization. A hysterectomy is generally required and is technically challenging, whereas removal of placenta percreta involves partial removal of the bladder wall with reconstruction. Therefore, the rate of urologic complications is higher compared to hysterectomy indicated for other reasons. 

Unlike prior reported data, we do not report a higher risk of urologic complications with CS performed for arrest of descent in the second stage of labour, cephalopelvic disproportion, breech presentation, marginal placenta previa, or uterine rupture [3,12,15,16]. In other words, the risk of urologic injuries is not increased in emergency CS performed for routine obstetrical indications. For obvious reasons, we cannot affirm that these complications cannot occur at routine CS, but we emphasize that these obstetrical indications are not major risk factors for urological complications.

Gestational age at delivery was a risk factor for urologic injury in our study. This can be explained by the fact that in cases with placenta accreta, the CS is perform earlier, in order to avoid the spontaneous onset of labour. We believe this may be the outcome of planning of elective surgeries earlier in pregnancy.

We did not note higher rates of urologic injuries when the surgery was performed by a young specialist compared to a consultant obstetrician. While this might be somehow expected, it is difficult to extrapolate and generalize from this finding, as this may represent the characteristics of the staff at our tertiary unit and how we organize the surgical teams for suspected high risk surgical cases. Nonetheless, this does point indirectly to the necessity of appropriate education for all future gynaecologic surgeons regarding risk-lowering procedural steps during hysterectomy. The findings from our study suggest that the potential for urologic injury should be assessed prior to the surgery, and the obstetric surgeon should elicit increased intraoperative awareness for prompt diagnosis and management. Involvement of a urology specialist when the lesion is suspected, along with evaluation strategies such as direct visualization at the time of injury occurrence, cystoscopy, and dye tests, have led to early diagnosis with adequate and timely management and improved outcomes. We are aware that having a urology specialist may at hand not be always possible, particularly for independent maternity hospitals that do not have other specialties nearby, physically located in the same institution. Thus, we advocate for maintaining the urology rotation for our residency program training. We also recognize the potential for missed cases, owing to late presentation to an outside facility. However, we consider the likelihood of such presentation as low, as we interrogated the presentations in the urology hospital, and our institution is the main tertiary referral centre for the regional obstetric care.

The increased size of the study population and the contemporary data represent main strengths of our study. The high number of CS performed, which reached a percentage of 60% of all deliveries at our centre, offers an advantage for a study of surgical complications, particularly through the high number of repeat CS. Thus, we believe we have representative data and are well positioned to report on the matter at hand. Furthermore, despite not having other specialty services at our unit location, we are fortunate to have a team of urology colleagues on site, able to recognize, help, and/or undertake care of patients with urologic complications. Nonetheless, this type of complication has a low incidence, and the small number of cases precluded us from further meaningful comparisons and statistical analyses. It is important to mention, however, that the overall incidence of urologic complications remained low, but rather constant, in the reports from the literature, ranging from 0.08 to 0.94%, in spite of expectations of seeing a rise over the past decade due to the increase in the rate of CS. In addition, in this study, we were able to provide details of the urologic injuries, their mode of occurrence, and severity, information that is frequently lacking in the previously published studies. 

Other limitations of our study include the retrospective design and the short-term follow-up post-surgery of only two weeks after CS. This time interval was chosen, as this was the maximum length of patient hospitalization after delivery. 

## 6. Conclusions

In conclusion, we report an incidence of urologic injuries at the CS surgery similar to previous literature reports, despite the substantial increase in the rate of CS. Our study shows that the risk of bladder and ureter injury is higher in patients with previous CS and associated complete placenta previa and/or accreta that require haemostatic hysterectomy. We recommend advanced preparation for these obstetrical cases, with an interdisciplinary surgical team consisting of a gynaecologist and urologist to attend and perform the surgery. Urologist involvement is instrumental in order to actively search for and solve potential urological complications.

We believe that a thorough assessment of women with previous CS, with an early US scan of the placenta location and relationship with the previous CS scar, may allow creation of a profile for the patients at risk for urological complications at the time of CS in order to organize the details of surgical management in a timely way and mitigate the risks of surgical morbidity.

## Figures and Tables

**Table 1:** Demographic and clinical characteristics of study population.

KERRYPNX	Cases—Urologic Injury (*n* = 36)	Controls—No Urologic Injury (*n* = 102)	*p*-Value
Age (years)	32.4 ± 4.3	28.8 ± 6.4	<0.01
Medium of origin			0.98
Urban	19 (52.8%)	54 (52.9%)	
Rural	17 (47.2%)	48 (47.1%)	
BMI (kg/m^2^)	29.90 ± 5.69	27.97 ± 4.5	0.16
Education			0.21
No education/unknown	2 (5.6%)	0 (0.0%)	
Less than high school	2 (5.6%)	21 (20.6%)	
High school	16 (44.4%)	44 (43.1%)	
Secondary education	16 (44.4%)	37 (36.3%)	
Socioeconomic status			0.39
medium and high income	34 (94.4%)	99 (97.1%)	
low income	2 (5.6%)	3 (2.9%)	
Gravidity (median, IQR)	3 (2, 4)	2 (1, 3)	0.003
Parity (median, IQR)			<0.001
0	4 (11.1%)	2 (1.96%)	
1	3 (8.3%)	46 (45.09)	
2	29 (80.6%)	54 (52.94%)	
Gestational age at delivery (weeks)	36.9 ± 1.5	37.9 ± 2.1	<0.01
Birthweight (gm)	2938.5 ± 476.5	3207.1 ± 667.8	<0.01
Apgar Score at 5 min	7.9 ± 0.7	7.9 ± 1.6	0.78

**Table 2:** Surgical characteristics of patients with urologic injury (cases) in comparison with patients with no urologic injuries (controls).

	Urologic Injury Cases (*n* = 34)	No Urologic Injury Controls (*n* = 102)	*p*-Value
Type of caesarean section (CS)			
Emergency CS	16 (44.4)	64(62.7)	0.55
Elective CS	20 (55.6)	38(37.25)	
Time of rupture of membranes			0.64
During CS	15 (41.6)	48 (47.0)	
Prior to CS	19 (58.4)	54 (52.9)	
Indication for CS			
Foetal distress	0	11	<0.01
Foetopelvic disproportion	0	9	<0.01
Marginal and lateral placenta praevia	4 (11.1)	20 (19.6)	<0.01
Complete placenta praevia	15 (41.7)	1 (1.0)	<0.01
Foetal presentation			
Abnormal presentation	5 (13.9)	9 (8.8)	0.52
Cephalic presentation	31 (86.1)	93 (91.2)	
Uterine rupture			
Yes	3 (8.3)	9 (8.8)	0.99
No	31 (91.7)	93 (91.2)	
Unsuccessful trial of labour	4	14	0.88
Placenta accreta spectrum disorder			
Yes	21 (58.3)	0 (0.0)	<0.01
No	15 (41.7)	102 (100.0)	
Uterine scar from previous CS	6	38	0.33
Number of previous CS			<0.001
0	4 (11.1)	63 (61.8)	
1	22 (61.1)	34 (33.3)	
≥2	10 (27.8)	5 (4.9)	
Pre-existing medical conditions	2 (6.0)	11 (10.0)	0.39
Pelvic adhesions			0.001
Yes	19 (52.8)	39 (38.2)	
No	17 (47.2)	63 (61.8)	
Type of anaesthesia at CS			<0.001
General	28 (77.7)	4 (4.0)	
Spinal	8 (22.3)	98 (96.0)	
Caesarean hysterectomy			<0.001
Yes	27 (75.0)	1 (1.0)	
No	9 (25.0)	101 (99.0)	
Adnexectomy			<0.001
Yes	10 (27.7)	0 (0.0)	
No	26 (72.2)	102 (100.0)	
Ligation of hypogastric arteries			<0.001
Yes	6 (16.7)	0 (0.0)	
No	30 (83.3)	102 (100.0)	
Intraoperative blood loss (mean ± SD) gm/100 mL	2.1 ± 1.2	0.81 ± 0.4	<0.01
Hb levels before CS (mean ± SD) g/dL	11.9 ± 0.7	12.02 ± 1.0	0.60
Hb levels after CS (mean ± SD) g/dL	9.6 ± 1.4	11.4 ± 2.3	<0.001
Difference between Hb levels before and after CS surgery	2.3 ± 1.4	0.6 ± 2.3	<0.001
Level of qualification of the surgeon			0.51
Consultant	35 (97.2)	96 (94.1)	
Specialist	1 (2.8)	6 (5.9)	

**Table 3:** Urological injuries occurring during CS: site, diagnosis, and management.

	Bladder Injury *n* = 32	Ureter Injury *n* = 4
Intraoperative assessment and diagnosis of urologic injury		
	32	2
Urologist intraoperative consult at CS	31	1
Direct vision	25	1
Dye infused (methylene blue)	7	
Cystoscopy (after surgery)	2	
Post-operative assessment and diagnosis of injury		
	1	3
Urine leakage through fistula	1	
Uroperitoneum		1
US/CT		1
Cystoscopy	1	
Fluoroscopy		
IVP		1
Type of injury		
Partial obstruction		2
Tear/laceration	32	2
Mechanism of injury		
Ureteral obstruction by suture		1
Ureteral kinking through tight neighbourhood sutures		1
Ureteral transection (partial or complete)		2
Bladder cutting/laceration during entry into the peritoneal cavity		
Bladder cutting/laceration during creation of the bladder flap	25	
Bladder cutting/laceration during uterine incision or delivery of the foetus	6	
Bladder included in the suture of the uterine incision, which developed into secondary vesicovaginal fistula	1	
Anatomic site of injury		
Bladder dome	27	
Posterior wall of the bladder	5	
Bladder trigone	0	
Pelvic ureter		4
Treatment		
Bladder suture	31	
Intraoperative removal of the transection sutures through the bladder	1	
Endoscopic stent		2
Immediate reimplantation of ureter		1
Surgical exploration and ureteral reimplantation		1
Postoperative recovery		
Duration of nephrostomy and ureteric stent		3 women—3 months 1 woman—6 months
Additional surgery	Partial cystectomy with bilateral JJ stent placement	

## Data Availability

The datasets used during the current study are available from the corresponding author on reasonable request.
